# Higher HEI-2015 Scores Are Associated with Lower Risk of Sleep Disorder: Results from a Nationally Representative Survey of United States Adults

**DOI:** 10.3390/nu14040873

**Published:** 2022-02-19

**Authors:** Ming-Gang Deng, Jia-Qi Nie, Yuan-Yuan Li, Xue Yu, Zhi-Jiang Zhang

**Affiliations:** 1 Department of Epidemiology, School of Public Health, Wuhan University, Wuhan 430071, China; 2016302170042@whu.edu.cn (M.-G.D.); 2021103050012@whu.edu.cn (Y.-Y.L.); 2019203050009@whu.edu.cn (X.Y.); 2 Department of Nutrition and Food Hygiene, School of Public Health, Wuhan University, Wuhan 430071, China; 2020203050021@whu.edu.cn

**Keywords:** sleep disorder, dietary quality, healthy eating index

## Abstract

Whether there is an association between dietary quality and sleep disorder in American adults is unclear. We conducted this study to analyze whether dietary quality, using the Healthy Eating Index-2015 (HEI-2015) scores as the measure, was associated with self-reported sleep disorders. Data came from the National Health and Nutrition Examination Survey (2005–2014). Step-weighted logistic regression models were performed to explore the relationships between the HEI-2015 scores and sleep disorder. Weighted quantile sum regression model was used to identify the HEI-2015 components most strongly associated with sleep disorders. According to quartiles, HEI scores were categorized into inadequate (<25%), average (25%–75%), and optimal (>75%). Compared to inadequate HEI status, average HEI status (OR: 0.961, 95%CI: 0.959–0.962) and optimal HEI status (OR: 0.913, 95% CI: 0.912–0.915) were associated with reduced risk of sleep disorder after multivariable adjustments. Greens and beans, added sugars, saturated fats, total vegetables and total protein foods were the top five important components for sleep disorders. Our results suggest that there is a statistically significant association between better dietary quality and reduced risk of sleep disorder among United States adults.

## 1. Introduction

Sleep disorders are a group of syndromes characterized by disturbance in the patient’s amount of sleep, quality or timing of sleep, or in behaviors or physiological conditions associated with sleep. Insomnia, sleep apnea, and circadian rhythm sleep–wake disorders are typical sleep disorders. More than one-third of adults experience transient insomnia at some point in their life, and circadian rhythm sleep–wake disorders are common [1]. In the general middle-aged population, moderate-to-severe sleep apnea can be found in about 30–50% of men and 11–23% of women [2,3].

Sleep disorder is an important public health issue that has been confirmed to be associated with many adverse health outcomes, such as obesity [4], hypertension [4], cardiovascular disease [4,5], premature mortality [6,7,8], and impaired quality of life [9].

Many studies have confirmed that nutrients, such as macronutrients, vitamins, etc., are related to sleep. For example, intake of fats was negatively associated with total sleep time [10], vitamin D deficiency was associated with a higher risk of sleep disorders [11], and vitamin C and selenium were found to be related to sleep duration [12]. In addition, high carbohydrate diets were suggested to improve sleep outcomes by the previous review [13]. However, the impact of overall diet quality is inconclusive because the health outcome of diet is often driven by the joint effect of all dietary intakes. For example, no associations between dietary scores and sleep parameters were found in Swedish older men [14]. American adults with higher energy-adjusted dietary inflammatory index were more likely to report sleep disturbances [15]. Moreover, the previous review pointed out that no sufficient evidence can support the conclusion that it is possible to improve sleep quality through interventions on dietary habits or on individual components of the diet [16].

The Healthy Eating Index-2015 (HEI-2015) was designed by the United States Department of Agriculture to measure how well a set of foods aligns with key recommendations of the 2015–2020 Dietary Guidelines for Americans.

We aim to explore the effect of overall diet quality in a more macroscopic way, using HEI-2015 as the measure, to determine whether general American adults with sleep disorders can benefit from adherence to American dietary guidelines.

## 2. Materials and Methods

### 2.1. Data Source

The participants who engaged in the 2005–2014 National Health and Nutrition Examination Survey (NHANES) were used in this study. NHANES, a major program of the National Center for Health Statistics (NCHS), is designed to assess the health and nutritional status of adults and children in the United States. The survey combined interviews and physical examinations, having high representation in the US population. More information can be found at https://www.cdc.gov/nchs/nhanes/ (accessed on 24 July 2021).

### 2.2. Participants

**Figure 1:**
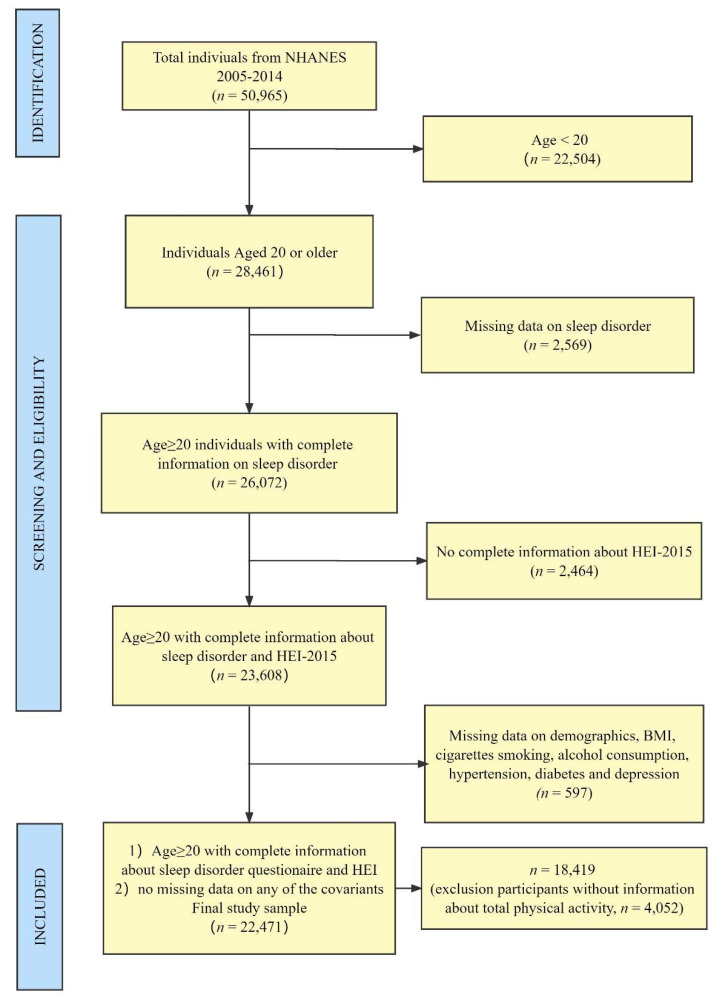
Flowchart of the population included in our final analysis.

Figure 1 describes the process of the study design, sampling, and exclusion. The study combined data from 2005 to 2014 NHANES cycles (i.e., 2005–06; 2007–08, 2009–10, 2011–12, 2013–14), and 28,494 participants were excluded because of the exclusion of participants aged less than 20 and missing data on any of the measures or covariates. In the NHANES, participants aged less than 20 years old were treated as a youth by education level. The average age of the participants was 47.5, of which 47.2% were men.

Questions about sleep habits and sleep disorders were added to the NHANES questionnaire beginning in 2005. Since 2015, we could not obtain information about sleep disorders, because of the updated questionnaires; thus, we only chose the data from 2005 to 2014 for this study.

### 2.3. Measures

#### 2.3.1. Sleep Disorder

Sleep disorder: In NHANES, sleep disorder was measured in the “Sleep Disorders Questionnaire”, where respondents are asked “Have you ever been told by a doctor or other health professional that you have a sleep disorder?”. If the respondent answered “Yes”, s/he was classified as “adults with sleep disorder”, or if the respondent answered “No”, s/he was classified as “adults without sleep disorder”.

#### 2.3.2. Healthy Eating Index 2015

Previous studies have assessed the HEI-2015 of construct validity, reliability, criterion validity, and so on, and concluded that it was a useful means by which to measure diet quality [17,18,19]. Moreover, HEI-2015 has been applied to the study of sleep duration [20], cardiovascular disease [21], and cognitive performance [22].

HEI-2015 is composed of nine adequacy components and four moderation components. Adequacy components represent the food groups, subgroups, and dietary elements that are encouraged. For these components, higher scores reflect higher intakes, because higher intakes are desirable. Moderation components represent the food groups and dietary elements for which there are recommended limits to consumption. For moderation components, higher scores reflect lower intakes because lower intakes are more desirable. The adequacy components are total fruits, whole fruits, total vegetables, greens and beans, whole grains, dairy, total protein foods, seafood and plant proteins, and fatty acids (ratio of unsaturated to saturated fatty acids). The moderation components are refined grains, sodium, added sugars, and saturated fats [23].

Participants who had both two 24 h dietary recalls were included, and the dietary recall status was restricted to be reliable or had met the minimum criteria for each day, which can reduce the bias of HEI-2015. The HEI-2015 total and component scores were calculated using the simple HEI scoring algorithm using publicly available SAS macros.

The HEI-2015 is scored out of 100 points, with higher scores indicating better overall diet quality. According to the quartiles, we divided the HEI scores into three groups: <25% (inadequate, reference group), 25–75% (average), and >75% (optimal), as HEI Category.

### 2.4. Covariates

Covariates of interest consisted of sociodemographic, behavioral, and health characteristics deemed a priori as potential confounders. Sociodemographic characteristics included age group (20–39, 40–59, 60+), sex, race (non-Hispanic White, Mexican American, Non-Hispanic Black, other race/multiracial), highest level of education (<High School, High school /GED equivalent, College/AA degree and College or above), and ratio of household income to the federal poverty level (FPL; 0~130% FPL, >130~350% FPL, >350% FPL). Behavioral characteristics included smoking status (never, former, current) [24], drink level (none, light, moderate and heavy) [25], and caffeine intake categories (<Q_1_, Q_1_~Q_3_ and >Q_3_). The categories of caffeine intake were defined by quartiles of the mean amounts of two 24 h dietary recalls. Health characteristics included body mass index categories (underweight, normal weight, overweight and obese) [26], hypertension (Yes, No) [27,28], diabetes (Yes, No) [27,29] and depression(Yes, No) [30,31].

### 2.5. Statistical Analysis

To make nationally representative estimates, a full sample 2-year Mobile Examination Center exam weight was used to account for different sampling probabilities and participation rates across the ten-year period according to the NHANES data analysis tutorials.

Descriptive statistics were used to compare distributions of measures and covariates with survey waves and sleep disorder. Weighted mean was used to calculate the mean HEI scores across years. Rao–Scott Chi-square test was used to compare differences among groups. A series of step-weighted logistic regression models was performed to analyze the association between HEI scores and sleep disorder. No covariates were adopted in the crude model, sociodemographic characteristics were adopted in model I, model II was additionally adjusted for behavioral factors, and model III was additionally adjusted for health characteristics. Weighted quantile sum regression model (WQS Model) was used to identify the individual HEI-2015 components most strongly associated with sleep disorder while adjusting for risk factors. The receiver operating characteristic (ROC) curve was performed to examine the fitness of the WQS model, using the original database. Statistical analyses were performed using the R software (version 4.1.0, R Foundation for Statistical Computing). All statistical tests were two-sided, and significance was considered at *p* < 0.05.

In the sensitivity analysis, we added the total physical activity in the logistics regression model and the WQS model, as consistent total physical activity assessment in NHANES was conducted starting from 2007. Total physical activity was assessed from 2007–2014, which included work and recreational moderate and vigorous activity and was categorized as 0, 1–149, 150–299, and ≥300 min/week.

## 3. Results

### 3.1. Descriptive Analysis

**Figure 2:**
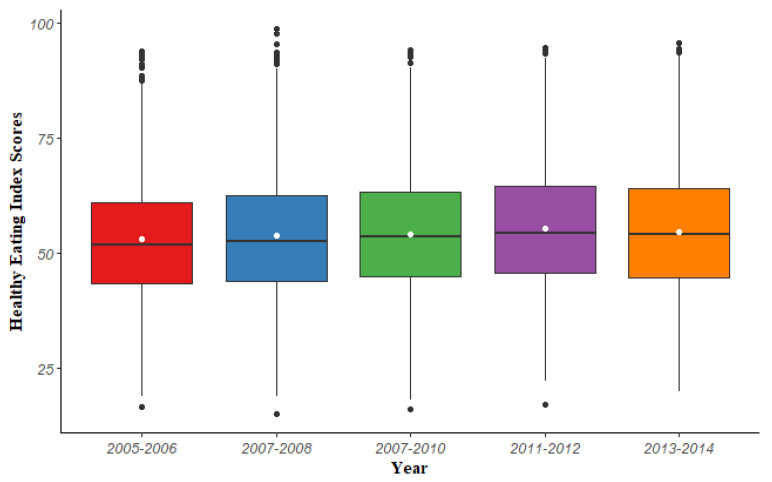
The trend of HEI-2015 scores in the five cycles from NHANES 2005–2014. The white points in the graph represent the weighted mean of the HEI-2015 scores for each study wave.

The overall mean and standard error of HEI-2015 scores were 54.05 and 0.21, respectively. This indicated that average diets of American adults do not conform to dietary recommendations. The mean of HEI scores rose from 52.76 in 2005–2006 to 54.45 in 2013–2014 (Figure 2). More accurately, American adults’ diet quality showed a slight improvement over time from 2005–2012 but a small decrease during 2013–2014, and there is room for improvement.

Table 1 depicts the prevalence of sleep disorder and the characteristic of HEI across five consecutive NHANES cycles. During this decade, the prevalence of sleep disorders in US adults increased from 7.50% in 2005–2006 to 10.41% in 2013–2014, showing significant growth (*F* = 5.4848, *p* < 0.001). Additionally, the proportion of those who have inadequate HEI scores is decreasing, while the proportion of those who have optimal HEI scores are increasing, which indicates that dietary quality has some degree of improvement during this decade. This is consistent with the results in Figure 2.

Each of the potential confounders was associated with sleep disorders at *p* < 0.05; see Table 2. Older adults and males are more likely to report sleep disorders than not reporting, whereas younger adults and females were more likely to report no sleep disorders compared to reporting sleep disorders. People who have higher education levels and lower family income, abnormal weight, chronic diseases, and depression were more likely to report sleep disorders than not reporting. Participants who had higher HEI scores and higher intake of caffeine and alcohol were less likely to report sleep disorders than reporting.

### 3.2. Logistic Regression Models to Assess the Association between HEI-2015 Total Scores and Sleep Disorder

The results of step-weighted logistics regression models are illustrated in Table 3. Compared to the crude model, model I, and model II, the effect of the HEI-2015 on sleep disorders was reduced in Model III. However, this association did not alter, suggesting that a higher HEI score or better dietary quality may be associated with decreased risk of sleep disorders.

In the sensitivity analysis, compared to those with inadequate HEI status, people with optimal HEI status were less likely to report sleep disorders (OR: 0.995, 95% CI: 994–0.997), indicating that higher HEI scores were associated with the decreased risk of sleep disorders. Detailed information was presented in the Supplementary Table S1.

### 3.3. WQS Regression Model to Assess the Association between HEI-2015 Components and Sleep Disorder

In the WQS regression model, component values were quantiled and combined into a unidirectional weighted index, thereby reducing dimensionality and avoiding multi-collinearity. WQS provides a single overall effect estimate of the mixture, and individual components were ranked by their overall contribution to the index, indicating relative importance.

Adjusted for sociodemographic, behavioral and health covariates, the overall effect of 13 components on sleep disorder was protective (OR: 0.977, 95% CI: 0.964–0.990); see Table 4. The results of the WQS model were similar to the results of the logistic model.

**Figure 3:**
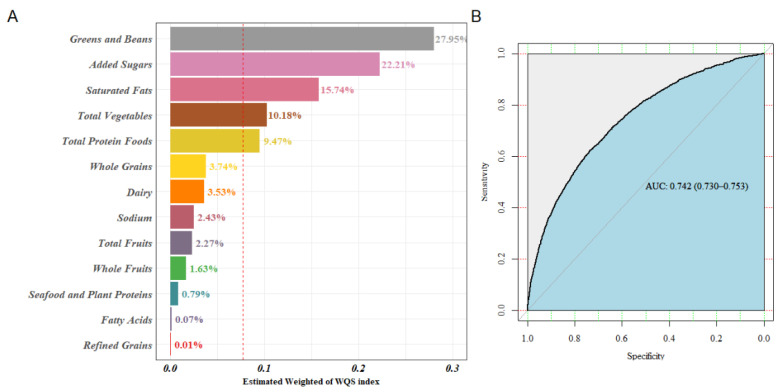
WQS regression index weights for sleep disorders. (**A**) ROC curve of the WQS model (**B**).

Figure 3A displays the WQS model regression index weights. Greens and beans, added sugars, saturated fats, total vegetables, and total protein foods were the top five important components, which account for more than 85% of the weight. Accordingly, a higher intake of greens and beans, total vegetables, and total protein foods, which were adequacy components, were related to a reduced risk of sleep disorder. Inversely, moderation components, with higher points awarded for lower consumption, such as added sugars and saturated fats, were positively associated with the risk of sleep disorder. Thus, people with sleep disorders should limit their intake of added sugars and saturated fats. The other six adequacy components, whole grains, dairy, total fruits, whole fruits, seafood, and plant proteins, accounted for 12% of the weights; therefore, they were important components that cannot be ignored. Nevertheless, sodium and refined grains moderation components, accounting for 3% of the weights, contributed much less to the overall effect.

The area under the curve was 0.742 (95% CI: 0.730–0.753), which indicated that the results of the WQS model were considered acceptable; see Figure 3B.

The results of sensitivity analysis are presented in Supplementary Table S2. When controlling for total physical activity, the association between the overall 13 components and sleep disorders remained inverse (OR: 0.987, 95% CI: 0.975–0.999), indicating the stability of our findings. The top five components associated with sleep disorders did not alter, and the detailed information about the WQS regression index weights of 13 components for sleep disorders is illustrated in Supplementary Figure S1.

## 4. Discussion

Our study found compelling evidence that dietary quality and dietary components, using HEI-2015 as the measure, were linked to sleep disorders, assessed by the statement, “ever told by a doctor or other health professional that they have sleep disorder”. A higher HEI score or better dietary quality may be correlated to the reduced risk of sleep disorder.

Despite that these were not fully consistent with the measurements of self-reported sleep disturbances, several previous studies showed a similar association between diet and sleep disorders. A case-control study targeted at Iranian adults found that people with greater adherence to the healthy dietary pattern were less likely to have primary insomnia [32]. Meanwhile, diet quality score was found to be associated with adequate sleep duration and reduced odds for short sleep duration in Australian women [33]. In American adults aged 45–84, adherence to the Mediterranean diet was less likely to report insomnia [34], and poor diet quality, characterized by the Alternative Healthy Eating Index-2010, was associated with moderate-to-severe obstructive sleep apnea [35]. Moreover, the energy-adjusted Dietary Inflammatory Index was found to be associated with the risk of short sleep duration and self-reported sleep disturbances in American adults [15]. However, we used different measurements of self-reported sleep disturbances from this work, where sleep disturbances were assessed by the statement, “ever told by a doctor or other health professional that you have trouble sleeping”. Additional meta-analyses showed that diet treatment can reduce the severity of obstructive sleep apnea [36].

In addition, some researchers deemed that individuals with sleep disorders may select an unhealthy diet. A prospective cohort study found that women with poor sleep quality were prone to intake more and had a lower-quality diet [37]. Some reviews pointed out that sleep influenced dietary choices; people who slept less were more likely to prefer fats, eat fewer vegetables, and choose irregular eating patterns [38,39]. The proposed mechanisms by which sleep may stimulate food intake were by the upregulation of the activity of the central nervous hypocretin system and changes in key appetite hormones [40]. However, since the data are cross sectional, the causal relationship between HEI and sleep disorders remains unclear in our study.

Greens and beans, total vegetables, total protein foods adequacy components and added sugars and saturated fats moderate components were the top five dietary components associated with sleep disorder in our study. Previous studies have found that short sleep duration, one kind of sleep disorder, was associated with poor diet quality, such as lower bean, protein, fruits, and whole grain intake [39,41], or low intake of vegetables and fish, and high intakes of confectionary foods [33]. Another cross-sectional study found that industrialized dietary pattern (high in sugar-sweetened beverages, fast foods) yielded higher odds for obstructive sleep apnea, compared with the traditional dietary pattern (high in legumes and tortillas) [42].

Possible mechanisms may explain these relationships. A cross-sectional study based on a Chinese sample found that vegetables and beans or bean products can reduce the risk of depression, which can influence sleep [43]. Foods such as plant species, including roots, leaves, fruits, and seeds, contain melatonin and serotonin. These foods may also contain tryptophan, which is a precursor for serotonin and melatonin [44]. However, the consumption of saturated fatty acids deteriorates sleep wellness, and total sleep time was negatively associated with intake of total fat and saturated fat [45]. Moreover, the systematic review found that the Healthy Eating Index showed better correlation with obesity [46], and dietary weight loss was effective in reducing the severity of obstructive sleep apnea. These factors may explain why a healthy diet is important for sleep.

Caffeine was generally considered to be bad for sleep [47], but in this study, it was contradictory. This may be due to interference from other covariates, such as education level and family income, as copious caffeine intake was a risk factor of sleep disorders before including these covariates. In addition, people with higher caffeine intake also had a higher prevalence of sleep disorders than those with lower caffeine intake. Another possible explanation is that caffeine is anti-inflammatory. If coffee is consumed earlier in the day, it is unlikely to influence sleep and can help lower systemic inflammation. The previous study found that, compared with non-drinkers, people who drank ≥4 cups of total coffee/day had lower concentrations of C-reactive protein and interleukin 6 (IL-6) [48], which are related to sleep disorders [49]. Caffeine may be helpful to sleep through the inflammatory pathway. However, we could not obtain the information about the time of coffee consumption.

The effect of the 13-component mixture in the WQS model was relatively smaller than HEI total effect in the logistic regression. The possible reason is that, in the WQS model, the values for the 13 components can be scored into quantiles by the median, and the odds ratio represents the comparison between higher than the median and lower than the median. However, in the logistic regression model, we divided HEI total scores into three groups and compared the optimal and inadequate scores, making the protective effect of HEI seem to be larger than that of the WQS model.

There are several limitations to this study. First, only adults were investigated in this study, making it difficult to generalize our findings to children and adolescents. Second, whether participants had a sleep disorder or not was measured by asking if they had ever been told by a doctor or health professional that they had a sleep disorder. This self-reported but physician-diagnosed sleep disorder may cause bias because no objective sleep measures were collected. Third, the HEI does not incorporate the timing of eating, which may be an important aspect to consider in future analyses on the effect of sleep disorder on diet quality. Last but not least, the causal relationship of HEI and sleep disorder cannot be inferenced because of the cross-sectional design.

In conclusion, within this sample of nationally representative American adults, our primary finding is that sleep disorder is rapidly growing in prevalence among American adults, which raises demand for public health interventions. More importantly, we discovered that sleep disorder was significantly associated with lower dietary quality.

## Figures and Tables

**Table 1:** Characteristics of adults aged 20+ years in NHANES 2005–2014.

Characteristics	2005–2006 (*n =* 4046)	2007–2008 (*n =* 4661)	2009–2010 (*n =* 5010)	2011–2012 (*n =* 4291)	2013–2014 (*n =* 4463)	*χ^2^*	*p* Value
**Sleep disorder (No. Weighted%)**	286 (7.50%)	384 (8.05%)	395 (7.65%)	382 (9.24%)	462 (10.41%)	5.48	<0.001
**HEI Category (No. Weighted%)**						3.64	0.003
Inadequate	1042 (27.28%)	1145 (26.29%)	1179 (23.22%)	942 (21.76%)	1093 (24.73%)		
Average	2129 (51.63%)	2373 (50.08%)	2610 (51.38%)	2158 (50.36%)	2149 (47.90%)		
Optimal	875 (21.09%)	1143 (23.63%)	1221 (25.40%)	1191 (27.88%)	1221 (27.37%)		

HEI, Healthy Eating Index.

**Table 2:** Characteristics of adults aged 20+ years by sleep disorder.

Characteristics	Adults without Sleep Disorder	Adults With Sleep Disorder	*χ^2^*	*p* Value
**HEI Category (No. Weighted%)**			4.02	0.019
Inadequate	4881 (90.60%)	520 (9.40%)		
Average	10467 (91.29%)	952 (8.71%)		
Optimal	5214 (92.44%)	437 (7.56%)		
**Age Group (No. Weighted%)**			56.40	<0.001
20–39	6800 (95.10%)	342 (4.90%)		
40–59	6766 (89.67%)	785 (10.33%)		
60+	6996 (89.39%)	782 (10.61%)		
**Gender (No. Weighted%)**			5.89	0.018
Female	10841 (91.97%)	938 (8.03%)		
Male	9721 (90.79%)	971 (9.21%)		
**Race (No. Weighted%)**			19.97	<0.001
Non-Hispanic White	9504 (90.71%)	1040 (9.29%)		
Mexican American	3260 (95.39%)	175 (4.61%)		
Non-Hispanic Black	4347 (91.43%)	417 (8.57%)		
Other	3451 (93.00%)	277 (7.00%)		
**Education Level (No. Weighted%)**			2.89	0.045
<High School	5141 (92.20%)	428 (7.80%)		
High school /GED	4711 (90.72%)	461 (9.28%)		
College/AA degree	5966 (90.77%)	615 (9.23%)		
College or above	4744 (92.20%)	405 (7.80%)		
**Family Income (No. Weighted%)**			3.47	0.035
0~130% FPL	5807 (90.12%)	636 (9.88%)		
130%~350% FPL	7108 (91.54%)	626 (8.46%)		
350% FPL	7647 (91.85%)	647 (8.15%)		
**Smoke Status (No. Weighted%)**			27.61	<0.001
Never Smoker	11450 (92.91%)	843 (7.09%)		
Former Smoker	5031 (89.26%)	605 (10.74%)		
Current Smoker	4081 (89.99%)	461 (10.01%)		
**Drink Level (No. Weighted%)**			16.11	<0.001
None	6377 (89.54%)	700 (10.46%)		
Light	5883 (90.47%)	598 (9.53%)		
Moderate	6566 (93.26%)	476 (6.74%)		
Heavy	1736 (92.43%)	135 (7.57%)		
**Caffeine Category (No. Weighted%)**			4.08	0.019
<Q_1_	6310 (91.72%)	524 (8.28%)		
Q_1_~Q_3_	10556 (91.85%)	928 (8.15%)		
>Q_3_	3696 (90.19%)	457 (9.81%)		
**BMI Category (No. Weighted%)**			80.31	<0.001
Normal Weight	5655 (95.60%)	256 (4.40%)		
Underweight	337 (94.46%)	12 (5.54%)		
Overweight	6981 (93.93%)	453 (6.07%)		
Obese	7589 (85.88%)	1188 (14.12%)		
**Hypertension (No. Weighted%)**			196.81	<0.001
No	13595 (93.88%)	825 (6.12%)		
Yes	6967 (86.12%)	1084 (13.88%)		
**Diabetes (No. Weighted%)**			157.57	<0.001
No	18299 (92.43%)	1449 (7.57%)		
Yes	2263 (81.09%)	460 (18.91%)		
**Depression (No. Weighted%)**			183.08	<0.001
No	17831 (92.71%)	1341 (7.29%)		
Yes	2731 (82.41%)	568 (17.59%)		

HEI, Healthy Eating Index; FPL, family income to poverty; BMI, Body Mass Index.

**Table 3:** Relationship between HEI and sleep disorder among US adults aged 20+ years.

Characteristics	OR (95% CI)			
	Crude Model	Model I	Model II	Model III
**HEI Category (reference, Inadequate)**				
Average	0.920 (0.919,0.921)	0.875 (0.874,0.876)	0.907 (0.906,0.909)	0.961 (0.959,0.962)
Optimal	0.789 (0.787,0.790)	0.720 (0.719,0.721)	0.771 (0.770,0.772)	0.913 (0.912,0.915)
**Age Group (reference, 20–39)**				
40–59		2.365 (2.361,2.368)	2.255 (2.252,2.258)	1.706 (1.704,1.709)
60+		2.460 (2.456,2.463)	2.192 (2.188,2.195)	1.468 (1.465,1.470)
**Sex (reference, Female)**				
Male		1.185 (1.183,1.186)	1.227 (1.226,1.229)	1.344 (1.342,1.346)
**Race (reference, Non-Hispanic White)**				
Mexican American		0.529 (0.528,0.530)	0.549 (0.548,0.551)	0.546 (0.545,0.548)
Non-Hispanic Black		0.915 (0.913,0.917)	0.903 (0.901,0.904)	0.728 (0.726,0.729)
Other		0.827 (0.825,0.828)	0.812 (0.811,0.814)	0.809 (0.808,0.811)
**Education Level (reference, <High School)**				
High school /GED equivalent		1.202 (1.199,1.204)	1.256 (1.254,1.259)	1.360 (1.358,1.363)
College/AA degree		1.319 (1.317,1.321)	1.416 (1.414,1.419)	1.522 (1.520,1.525)
College or above		1.143 (1.140,1.145)	1.331 (1.328,1.333)	1.682 (1.679,1.686)
**Family Income (reference, 0~130% FPL)**				
130%~350% FPL		0.715 (0.714,0.716)	0.744 (0.743,0.745)	0.841 (0.84,0.842)
350% FPL		0.645 (0.644,0.646)	0.705 (0.704,0.706)	0.871 (0.87,0.873)
**Smoke Status (reference, Never Smoker)**				
Former Smoker			1.437 (1.435,1.439)	1.346 (1.345,1.348)
Current Smoker			1.439 (1.436,1.441)	1.509 (1.507,1.511)
**Drink Level (reference, None)**				
Light			0.955 (0.953,0.956)	0.991 (0.990,0.993)
Moderate			0.617 (0.616,0.618)	0.752 (0.751,0.754)
Heavy			0.754 (0.753,0.756)	0.411 (0.41,0.412)
**Caffeine Category (reference, <Q_1_)**				
Q_1_~Q_3_			0.900 (0.899,0.901)	0.901 (0.900,0.902)
>Q3			0.886 (0.884,0.887)	0.865 (0.864,0.867)
**BMI Category (reference, Normal Weight)**				
Underweight				1.254 (1.248,1.261)
Overweight				1.241 (1.239,1.243)
Obese				2.733 (2.728,2.737)
**Hypertension (reference, No)**				
Yes				1.605 (1.603,1.607)
**Diabetes (reference, No)**				
Yes				1.616 (1.613,1.618)
**Depression (reference, No)**				
Yes				3.365 (3.360,3.370)

OR, odds ratio; 95% CI, 95% confidence interval; HEI, Healthy Eating Index; BMI, Body Mass Index. Model I: Adjusted for sociodemographic characteristics. Model II: Adjusted for sociodemographic characteristics and behavioral characteristics. Model III: Adjusted for sociodemographic characteristics, behavioral characteristics, and health characteristics.

**Table 4:** The results of the weighted quantile sum regression model.

Model	OR	95% CI	*p* Value
Model I	0.963	(0.951,0.975)	<0.001
Model II	0.974	(0.961,0.986)	<0.001
Model III	0.977	(0.964,0.990)	<0.001

OR, odds ratio; 95% CI, 95% confidence interval. Model I: Adjusted for sociodemographic characteristics. Model II: Adjusted for sociodemographic characteristics and behavioral characteristics. Model III: Adjusted for sociodemographic characteristics, behavioral characteristics, and health characteristics.

## Data Availability

The NHANES dataset is publicly available online, accessible at cdc.gov/nchs/nhanes/index.htm.

## Supplementary Materials

The following supporting information can be downloaded at: https://www.mdpi.com/article/10.3390/nu14040873/s1, Table S1: The sensitivity analysis results of the logistics regression model from 2007-2014; Table S2: The sensitivity analysis results of the Weighted Quantile Sum regression model; Figure S1: The sensitivity analysis results of (A) WQS regression index weights for sleep disorders; (B) The ROC curve of the WQS model.
