# Supplementary material for: Higher HEI-2015 Scores Are Associated with Lower Risk of Sleep Disorder: Results from a Nationally Representative Survey of United States Adults
Source: Nutrients. 2022 Feb 19;14(4):873. doi: 10.3390/nu14040873 (PMC8876288; doi:10.3390/nu14040873)
Supplement: Supplementary file 1 [file nutrients-14-00873-s001.zip › nutrients-1530973-Supplementary.pdf]

Table S1 The sensitivity analysis results of the logistics regression model from 2007-2014

Characteristics	OR (95% CI)			
	Crude Model	Model I	Model II	Model III
**HEI Category (reference, Inadequate)**				
Average	0.947(0.946-0.948)	0.917(0.916,0.918)	0.964(0.962,0.965)	1.013(1.012,1.015)
Optimal	0.809(0.808-0.810)	0.764(0.763,0.766)	0.861(0.860,0.862)	0.995(0.994,0.997)
**Age Group (reference, 20-39)**				
40-59		2.203(2.200,2.206)	2.014(2.012,2.017)	1.594(1.592,1.597)
60+		2.319(2.316,2.322)	1.841(1.838,1.844)	1.317(1.315,1.320)
**Sex (reference, Female)**				
Male		1.162(1.161,1.164)	1.269(1.267,1.27)	1.362(1.361,1.364)
**Race (reference, Non-Hispanic White)**				
Mexican American		0.495(0.493,0.496)	0.501(0.499,0.502)	0.499(0.498,0.500)
Non-Hispanic Black		0.936(0.934,0.937)	0.881(0.880,0.883)	0.740(0.739,0.741)
Other		0.797(0.795,0.798)	0.748(0.747,0.750)	0.769(0.768,0.771)
**Education Level (reference, < High School)**				
High school /GED equivalent		1.235(1.233,1.238)	1.369(1.366,1.371)	1.466(1.463,1.469)
College/AA degree		1.318(1.316,1.320)	1.516(1.513,1.518)	1.586(1.583,1.589)
College or above		1.064(1.062,1.066)	1.351(1.348,1.354)	1.643(1.640,1.646)
**Family Income (reference, 0~130% FPL)**				
130%~350% FPL		0.748(0.747,0.749)	0.799(0.798,0.801)	0.879(0.877,0.88)
350% FPL		0.677(0.676,0.678)	0.768(0.767,0.769)	0.911(0.909,0.912)
**Smoke Status (reference, Never Smoker)**				
Former Smoker			1.531(1.529,1.533)	1.432(1.430,1.433)
Current Smoker			1.517(1.515,1.520)	1.557(1.555,1.559)
**Drink Level (reference, None)**				
Light			0.96(0.959,0.9610)	1.004(1.003,1.006)
Moderate			0.633(0.632,0.634)	0.760(0.759,0.761)
Heavy			0.741(0.739,0.743)	0.435(0.434,0.436)
**Caffeine Category (reference, <Q1)**				
Q1~Q3			0.893(0.892,0.894)	0.896(0.895,0.897)
>Q3			0.836(0.834,0.837)	0.795(0.794,0.797)
**Total physical activity (reference, 0)**				
1-149			0.765(0.764,0.767)	0.876(0.874,0.878)
150-299			0.740(0.739,0.742)	0.884(0.882,0.885)
≥300			0.614(0.613,0.615)	0.767(0.766,0.768)
**BMI Category (reference, Normal Weight)**				
Underweight				1.218(1.212,1.224)
Overweight				1.207(1.205,1.209)
Obese				2.544(2.540,2.548)
**Hypertension (reference, No)**				
Yes				1.642(1.640,1.644)
**Diabetes (reference, No)**				
Yes				1.509(1.507,1.511)
**Depression (reference, No)**				
Yes				3.021(3.016,3.025)

Sample size = 18419; OR = Odds Ratio; 95% CI = 95% Confidence Interval; HEI = Healthy Eating Index; BMI = Body Mass Index. Model I: Adjust for sociodemographic characteristics. Model II: Adjust for sociodemographic characteristics and behavioral characteristics. Model III: Adjust for sociodemographic characteristics, behavioral characteristics, and health characteristics.

Table S2. The sensitivity analysis results of the Weighted Quantile Sum regression model

**Model**	**OR**	**95% CI**	***P*-Value**
Model I	0.975	(0.963,0.987)	<0.001
Model II	0.984	(0.972,0.996)	0.008
Model III	0.987	(0.975,0.999)	0.034

OR = Odds Ratio; 95% CI = 95% Confidence Interval. Model I: Adjust for sociodemographic characteristics. Model II: Adjust for sociodemographic characteristics and behavioral characteristics. Model III: Adjust for sociodemographic characteristics, behavioral characteristics, and health characteristics.

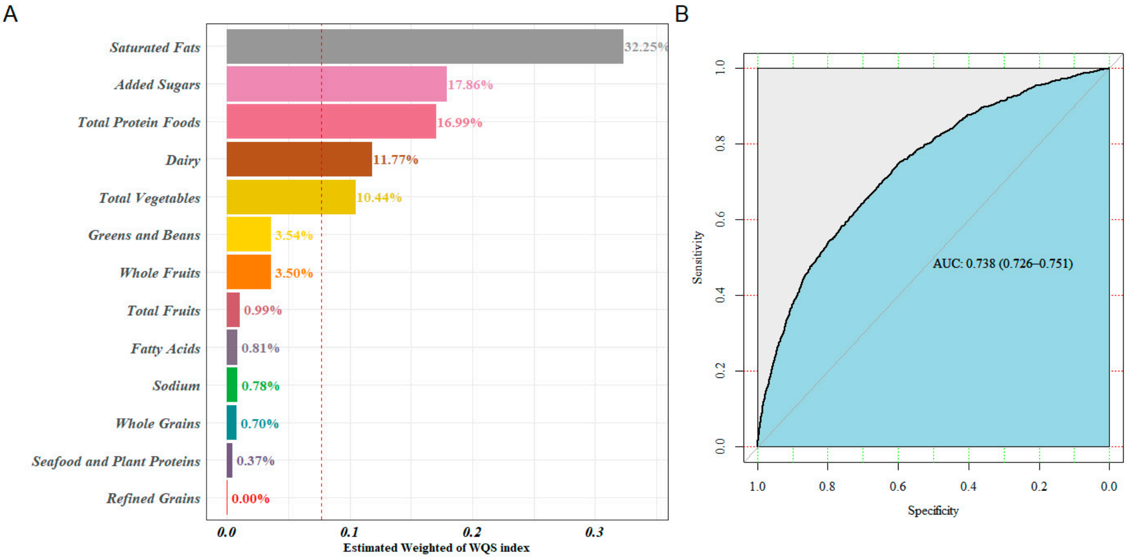

Figure S1 The sensitivity analysis results of (A) WQS regression index weights for sleep disorders; (B)The ROC curve of the WQS model.
